# Calcium Release-Activated Calcium Modulator ORAI1-Sensitive Serine Dehydratase Regulates Fatty Acid-Induced CD4^+^ Th17/Treg Imbalance in Dairy Cows

**DOI:** 10.3390/ani15030388

**Published:** 2025-01-30

**Authors:** Bingbing Zhang, Jingjing Wang, Ming Li, Jianan Wen, Juan J. Loor, Shuang Wang, Ziwei Ji, Xinquan Lv, Guihua Wang, Cheng Xia, Wei Yang, Chuang Xu

**Affiliations:** 1 College of Life Science and Technology, Heilongjiang Bayi Agricultural University, Daqing 163319, China; zhangbingbing613@gmail.com (B.Z.); 13359502976@163.com (J.W.); wenjianan8612@163.com (J.W.); jiziwei102623@163.com (Z.J.); wgh645@163.com (G.W.); 2 College of Animal Science, Ningxia University, Yinchuan 750021, China; wangshuang423@126.com; 3 College of Animal Science and Veterinary Medicine, Heilongjiang Bayi Agricultural University, Daqing 163319, China; liming5697@163.com (M.L.); lv102118@163.com (X.L.); xcwlxyf@sohu.com (C.X.); yangwei416@126.com (W.Y.); 4 College of Veterinary Medicine, China Agricultural University, Beijing 100193, China; 5 Mammalian Nutri Physio Genomics, Department of Animal Sciences and Division of Nutritional Sciences, University of Illinois, Urbana, IL 61801, USA; jloor@illinois.edu

**Keywords:** CD4^+^ T cell, serine dehydratase, ORAI1, high FFA, IL-17, FOXP3

## Abstract

High concentrations of free fatty acids (FFAs) caused by negative energy balance render the cow more prone to inflammatory diseases in part due to an imbalance in the types of immune cells and their specific functions. We previously demonstrated that ORAI calcium release-activated calcium modulator 1 (ORAI1) was associated with increased CD4^+^ Th17 content, but the precise mechanisms remain unclear. The purpose of this study was to evaluate the efficacy of FFAs on CD4^+^ T cell inflammatory response. High FFAs in dairy cows caused the transcript level of the pro-inflammatory factor *IL-17A*, plasma concentration of IL-17A, and amount of intracellular IL-17A to increase while the transcript levels and intracellular amount of the anti-inflammatory factor FOXP3 were downregulated. These changes indicated Th17/Treg imbalance and inflammation in dairy cows with high FFA. Moreover, ORAI1 and SDS abundance was elevated in dairy cows with high FFAs by transcriptomics, QPCR, and Western blot. Knockdown of SDS (siSDS) did not alter *ORAI1* expression in CD4^+^ T cells from high-FFA cows, while it decreased the expression of inflammatory factors. Transfection of CD4^+^ T cells using siRNA knockdown for ORAI1 (siORAI1) revealed that *SDS* and inflammatory factor abundance decreased. Serine can be catabolized to pyruvate by the action of serine dehydratase (SDS). Data from this study suggested that high FFAs caused by negative energy balance after calving regulates the Th17/Treg balance via SDS, but SDS does not regulate ORAI1 abundance. The above data suggested a pro-inflammatory mechanism in CD4^+^ T cells regulated by the ORAI1-sensitive SDS pathway in early postpartum cows experiencing high-FFA conditions. Thus, targeting this pathway may represent a new therapeutic and interventional approach for preventing immune-related disorders around parturition.

## Simple Summary

In this work, data suggested a pro-inflammatory mechanism in CD4^+^ T cells regulated by the ORAI1-sensitive SDS pathway in early postpartum cows experiencing high-FFA conditions. Thus, targeting this pathway may represent a new therapeutic and interventional approach for preventing immune-related disorders around parturition.

## 1. Introduction

Dairy cows with high free fatty acids (FFAs ≥ 1.2 mM) in the early post-calving period show metabolic disorders and stress, all of which highlight the disruption of physiological homeostasis [1,2]. As the periparturient cow’s intake decreases, the energy requirement for her lactation increases abruptly during this process, and this series of changes results in a negative energy balance in the body. Early lactation is a common period for the development of high FFAs in dairy cows [3,4]. CD4^+^ T cells play a vital role in the adaptive immune system and are involved in the pathogenesis of many diseases; a lot of research has clarified that metabolism imbalance interacts with immune disorders [5,6,7]. Several human and animal investigations have revealed significant variations and critical roles of Th17/Treg in chronic inflammation related with obesity and metabolic illness [8]. It has been reported that Th17 cells play an important role in liver inflammation, while Treg cells have anti-inflammatory functions and protect tissues from inflammation [9,10]. Serine dehydratase (*SDS*) catabolizes serine to pyruvate, a metabolite that is used by Th17 cells to produce acetyl coenzyme A via the tricarboxylic acid cycle in mouse CD4^+^ T cells [11]. Th17 cells induce systemic inflammation by producing IL-17A and IL-22 [12]. Treg cells produce IL-10, which helps to maintain peripheral immune tolerance [13]. Because the Th17/Treg balance is essential for maintenance of cellular homeostasis [14], understanding their role in the context of the early post-calving period in dairy cows is important.

In a previous study with dairy cows, we demonstrated that an abundance of store-operated Ca^2+^ entry moiety ORAI1 in CD4^+^ T cells was increased when cells were exposed to high concentrations of FFAs [15]. ORAI1 and stromal interaction molecule 1, major components of store-operated Ca^2+^ entry, are essential for Th17 cell growth and function [16]. Ca^2+^ influx in immune cells is regulated by activation of STIM1 and its Ca^2+^ channel ORAI1 [17]. As an important second messenger, intracellular Ca^2+^ is involved in cell proliferation, differentiation, and motility [18].

It has been reported that SDS affects pyruvate production and pyruvate is metabolized to generate acetyl-CoA, which in turn affects the content of Th17 cells in the body [11]. Thus, this study aimed to elucidate the mechanistic link between ORAI1-sensitive SDS and high-FFA-mediated CD4^+^ Th17/Treg imbalance in dairy cows.

## 2. Materials and Methods

This study was conducted according to the guidelines of the Declaration of Helsinki and was approved by Heilongjiang Bayi Agricultural University’s Ethics Committee for the Use and Care of Animals (approval no. SMKXJSXY2022003).

### 2.1. Animals and Sample Collection

Lactating Holstein cows (*n* = 12) from a 1000-cow dairy farm in Heilongjiang Province (China) were selected for this study [19,20]. The selected cows averaged 10 ± 1.90 d postpartum and 2.83 ± 0.42 lactations, had similar body conditions (3.10 ± 0.29), and had a normal calf delivery. They underwent a routine physical examination by the attending veterinarian to ensure the absence of other co-morbidities such as retained placenta, lameness, and subclinical mastitis [21,22]. Six cows with FFA levels above 1.2 mM and no clinical signs of disease were subsequently designated as high-FFA. Another 6 randomly chosen cows with FFA concentrations less than 0.8 mM and no signs of clinical illness were chosen as the healthy controls. The attending veterinarian screened all cows for BLV or other issues during a routine medical exam using the Bovine Leukemia Blocking Antibody Test Kit. After disinfection with iodine rub and 75% alcohol, before feeding, the tail vein was used to harvest 3 blood samples. A test tube containing heparin sodium anticoagulant (about 50 mL of blood per collection) was used to isolate CD4^+^ T cells. We stored the collected peripheral blood at 4 °C for the in vivo experiment and brought it back to the lab for cell separation within 2 h. Using a bovine peripheral blood lymphocyte isolation kit (Solarbio, Beijing, China), the lymphocytes were separated from the rest of the blood in accordance with the manufacturer’s protocols. The characteristics of the cows used are in Table 1.

For the in vitro experiment, in order to isolate CD4^+^ T cells, 4 healthy female Holstein calves (1 d old, 40–50 kg BW, fasting, rectal temperature 38.7 to 39.7 °C) from the above-mentioned dairy farm served as spleen donors. Subsequently, calves were euthanized humanely via intravenous injection of sodium thiosulphate. Fresh spleen parenchyma was removed aseptically and chopped into small pieces, the spleen sac was removed, and the spleen was pulverized to obtain splenocytes. After that, the spleen was washed twice for 10 min each time with PBS containing 5000 UI/mL of gentamicin and amphotericin. Cell suspensions from each calf were passed sequentially through cell sieves with mesh sizes of 50 (300 μm), 100 (150 μm), and 200 (75 μm). After two rounds of washing in RPMI-1640 alkaline medium (Cyclone Laboratories, Inc., Logan, UT, USA), the cell suspension was used to extract lymphocytes via centrifugation at 500× *g* for 5 min.

### 2.2. Isolation and Culture of CD4^+^ T Cells

Each calf’s (*n* = 3) lymphocyte preparation was centrifuged at 500× *g* for 5 min. CD4^+^ T cells were subsequently positively selected using MACS (Miltenyi Biotec, Bergish Gladbach, Germany) separation. Briefly, 1 × 10^7^ cells were labeled with 10 μg/mL MOUSE ANTI BOVINE CD4 (clone CC30, Bio-Rad AbD Serotec, Hercules, CA, USA) in 100 μL MACS Separation Buffer (Miltenyi Biotech, Bergish Gladbach, Germany) for 25 min at 4 °C. Then, Anti-Mouse IgG MicroBeads (Miltenyi Biotech, Bergish Gladbach, Germany) were used for magnetic labeling at 4 °C for 15 min in 100 μL MACS Separation Buffer. MS Separation Columns (Miltenyi Biotech, Bergish Gladbach, Germany) were used to separate the marked CD4^+^ T cells after washing twice with MACS Separation Buffer [23]. CD4^+^ T cells adsorbed on the column were used for subsequent analysis.

### 2.3. RNA Isolation and qRT-PCR

According to the manufacturer’s recommendations, total RNA was isolated using TRIzol (Invitrogen Company, Shanghai, China). RNA was dissolved in UltraPure distilled water (DNAse, RNase, Free). Using a PrimeScript RT reagent kit with gDNA Eraser (Takara Bio, Dalian, China), the RNA (1 μg) was reverse-transcribed into cDNA in accordance with the manufacturer’s instructions. The mRNA abundance was detected using an SYBR green plus reagent kit (Innovagene, Changsha, China). A total of 10 μL of SYBR Green Master, 2 μL of primers (one forward primer (10 μM) and one reverse primer (10 μM)), 2 μL of cDNA templates, and 6 μL of RNase-free distilled water made up the reaction system. Denaturation took place at 95 °C for 3 min, followed by 40 cycles of amplification (denaturation for 15 s at 95 °C, annealing for 1 min at 60 °C, and extension for 1 min at 60 °C) and extension for 5 min at 72 °C. The mRNA abundance of each target was normalized to glyceraldehyde-3-phosphate dehydrogenase (*GAPDH*) and *β-actin* (*ACTB*). The 2^−ΔΔCT^ method was used to measure abundance. The reactions were carried out using the Bio-Rad iCycler iQTM Real-Time PCR Detection System (Bio-Rad Laboratories Inc., Hercules, CA, USA). Table 2 contains a list of the primer sequences used for qPCR.

### 2.4. Protein Extraction and Western Blotting

CD4^+^ T cells were lysed with RIPA buffer (Beyotime Biotechnology, Shanghai, China) containing protease inhibitor on ice for 30 min, then centrifuged at 1400× *g* for 5 min at 4 °C. The concentration of protein was assessed with a BCA protein assay kit (Beyotime Biotechnology, Shanghai, China). Aliquots of 30 μg of total protein were loaded into 10% SDS–polyacrylamide gel electrophoresis together with established molecular weight markers (Solarbio, Beijing, China). Subsequently, the protein was electro-transferred to membranes made of polyvinylidene fluoride (Millipore Corp., Burlington, CA, USA). It was then enclosed in a containment solution (0.1% Triton-X/PBS containing 5% skimmed milk powder) for 1 h at room temperature. The blocked membranes were incubated overnight at 4 °C with specific antibodies for SDSL (1:1000, PHT8927, Abmart, Ephrata, PA, USA), ORAI1 (1:1000, 28411-1-AP, Proteintech, Rosemont, IL, USA), and β-actin (1:1000, sc-47778; Santa Cruz Biotechnology, Dallas, TX, USA). The membranes were then treated with secondary HRP-conjugated antibodies (3:5000; Beyotime Biotechnology) for 45 min at room temperature. Protein abundance signals could be detected with ECL (Beyotime Biotechnology, Shanghai, China)) using a protein simple imager. The band strength was measured using Imaging Lab software 1.8.0 (Miltenyi Biotech, Bergish Gladbach, Germany).

### 2.5. Transcriptomic Testing

Total RNA was isolated from CD4^+^ T cells in blood (*n* = 6 per group, 1 × 10^7^ cells per sample) using TRIzol (Invitrogen, Carlsbad, CA, USA). The quality of the RNA was then assessed using the NanoDrop ND-1000(Thermo Fisher Scientific, Waltham, MA, USA). The Agilent 2100 Bioanalyzer (Agilent Technologies, Santa Clara, CA, USA) was used to assess the integrity of the RNA. Sample RIN values were all >7.0. DynabeadsTM Oligo (dT) 25 (Invitrogen, Carlsbad, CA, USA) were used. First, two rounds of purified capture of poly(A) mRNA were performed. Afterwards, poly(A) mRNA was degraded at high temperatures using divalent cations to break it down into shorter fragments. Next, reverse transcriptase synthesized cDNA using the cleaved RNA. These DNA and RNA complex double strands were then converted into DNA double strands by the action of *E. coli* DNA polymerase I (*E. coli* DNA polymerase I) as well as RNase H. The DNA and RNA complexes were converted into DNA double strands. To give the double-stranded DNA a blunt end, DUTP was added to both strands simultaneously. An A base was then added to the blunt end of each double strand to help them attach to the adapter. Each node had a T-base protrusion that bound to the DNA fragment with an A end. Finally, the DNA fragments were screened for size and purified by magnetic beads. After treatment of U-labeled second-strand DNA with heat-unstable UDG enzyme, PCR was used to create libraries with fragment sizes of 250–350 bp. Finally, we performed 150 bp paired-end sequencing of Illumina Hiseq 4000 (LC Bio, Hangzhou, China) using the technology suggested by the vendor. Library creation and sequencing were conducted by Lane Crawford Biotech, China Ltd. (Hong Kong, China).

### 2.6. Treatment of siSDS or siORAI1

In vitro experiments were performed with isolated spleen CD4^+^ T cells from each individual calf. Before treatment with siSDS or siORAI1, 6-well plates contained seeds of cells at a density of 1 × 10^6^ cells/cm^2^ (2 mL per well) with RPMI-1640 medium supplemented containing 10% fetal bovine serum, 100 U/mL penicillin, and 100 μg/mL streptomycin with 5% CO_2_ for 24 h at 37 °C. For transient transfections, 1 × 10^6^ cells were seeded in 6-well plates for 48 h prior to the experiment. According to the manufacturer’s instructions, siRNA (Shanghai GenePharma Co., Ltd., Shanghai, China) was used to transfect cells with siORAI1/siSDS.

### 2.7. ELISA Analysis

Blood samples from the tail vein of the 12 cows were centrifuged at 1400× *g* for 5 min and the harvested serum was used for analyses within 2 h. In vitro assays were performed to collect treated CD4^+^ T cells for detection of intracellular IL-17 content (Lengton, Shanghai, China) utilizing bovine-specific ELISA kits and following the manufacturer’s directions.

### 2.8. Flow Cytometry

Spleen CD4^+^ T cells from each calf were re-stimulated with 50 ng/mL phorbol myristate acetate, 1 μM ionomycin, and 5 μM Brefeldin A for 4 h to label cytokines. CD4 Antibody (Invitrogen Corporation, China) and Fixable Viability Dye (Invitrogen Company, Shanghai, China) in PBS were used on the cell surface following the manufacturer’s recommendations when staining. Then, permeabilization was performed using the FoxP3/Transcription Factor Staining Buffer Set (eBioscience, Waltham, MA, USA). In addition, because CD4^+^ T cells were primary cells, variation in FoxP3 expression cells was expected and resulted in a gradient in its expression. Anti-Foxp3 (2 g/mL, clone FJK-16s; Invitrogen Corporation, China) and anti-IL-17A (0.25 g/mL, clone eBio17B7; eBioscience, Waltham, MA, USA) antibodies were employed. Negative flow cytometry controls included Isotype Control (17-4321-81, eBioscience, Waltham, MA, USA).

### 2.9. Statistical Analysis

Data are presented as the means ± SEM, where *n* is the number of independent experiments. The GraphPad Prism tool and SPSS 26.0 Software (IBM, Chicago, IL, USA) were used for statistical analysis (Prism 8.0; GraphPad Software, San Diego, CA, USA). The statistical significance was assessed using an unpaired Student *t*-test or one-way ANOVA with Bonferroni correction. Only differences with *p* ≤ 0.05 were deemed statistically significant, while *p* ≤ 0.01 was deemed highly significant.

## 3. Results

### 3.1. High FFAs Induced Elevated CD4^+^ Th17 Cell Production in Dairy Cows

**Figure 1:**
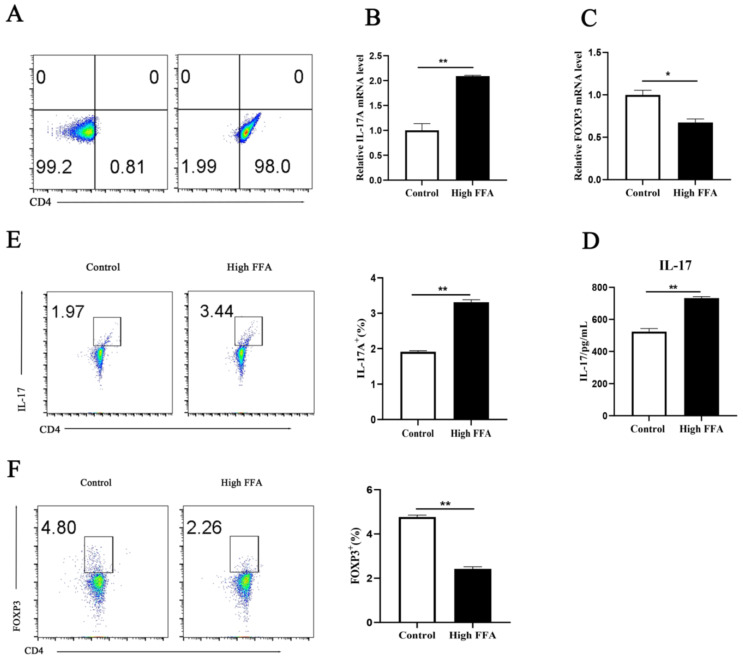
Isolation of CD4^+^ T cells from peripheral blood of healthy and high-FFA cows. (**A**) CD4^+^ T cell purification efficiency. (**B**,**C**) Relative levels of mRNA for *IL-17A* and *FOXP3*. (**D**) Concentrations of IL-17 in serum from control (*n* = 6) and high-FFA (*n* = 6) cows. (**E**) The content of IL-17A in cells was analyzed by flow cytometry. (**F**) The content of FOXP3 in cells was analyzed by flow cytometry. Independent samples t-test for comparison. The data presented are the mean ± SEM; ** *p* ≤ 0.01, * *p* ≤ 0.05.

Cell purity was checked after sorting peripheral blood CD4^+^ T lymphocytes. Purity reached more than 98% (Figure 1A); thus, this method was deemed suitable for subsequent experiments. Compared with the control group, the transcript level of the pro-inflammatory factor *IL-17A* (*p =* 0.0001), *IL-6* (*p* = 0.0810), *IFN-γ* (*p* = 0.0001) was upregulated and the transcript levels of the anti-inflammatory factor *FOXP3 (p =* 0.0015), *IL-10* (*p* = 0.0040), *TGF-β* (*p* = 0.0001) were downregulated in the blood CD4^+^ T cells from cows with high FFAs (Figure 1B,C, Supplementary Figure S1). ELISA results revealed greater levels of IL-17 (*p* = 0.0001) in the peripheral blood of cows with high FFAs (Figure 1D). Results using flow cytometry revealed upregulation of *IL-17A* (*p* = 0.0001) release from Th17 cells and downregulation of *FOXP3* (*p =* 0.0001) from CD4^+^ T cells in cows with high FFAs (Figure 1E,F).

### 3.2. Elevated SDS Expression in Cows with High FFAs

**Figure 2:**
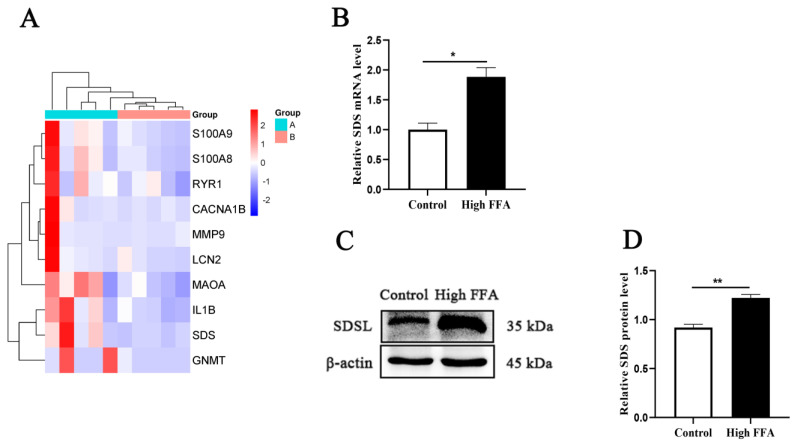
Expression of SDS in control and high-FFA cows. (**A**) Gene group analyses for high-FFA (Group A) and control (Group B) cows. (**B**) Relative mRNA abundance of SDS. (**C**) Western blots of SDSL. (**D**) Relative abundance protein content of SDSL. Independent samples t-test for comparison. The data presented are the mean ± SEM; ** *p* ≤ 0.01, * *p* ≤ 0.05.

Transcriptomic results indicated that expression of genes related to serine and valine metabolism were significantly upregulated in dairy cows with high FFAs (Figure 2A, Supplementary Figure S2). The transcript level of *SDS* (*p* = 0.0017) was significantly greater in dairy cows with high FFAs (Figure 2B). The protein abundance of SDS (*p* = 0.0002) was also significantly greater in dairy cows with high FFAs (Figure 2C,D).

### 3.3. Effect of Serine Dehydratase (SDS) on CD4^+^ Th17/Treg

**Figure 3:**
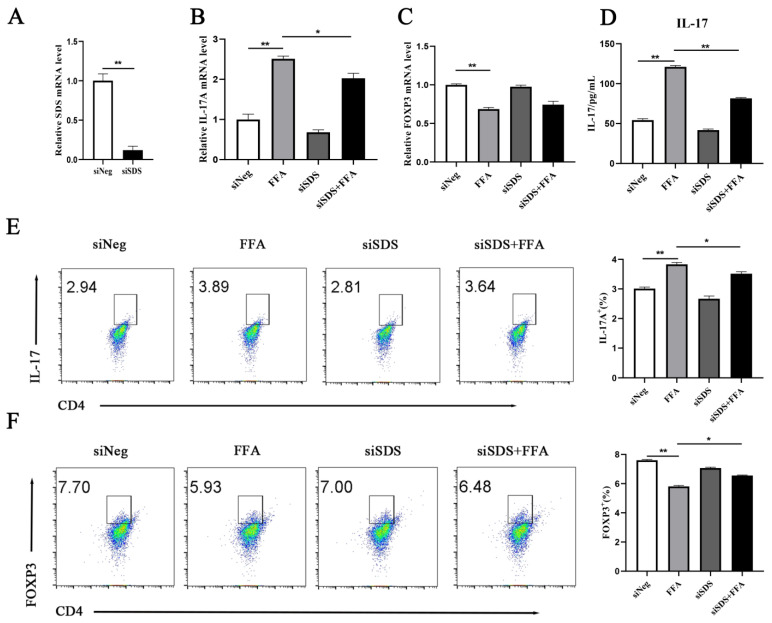
CD4^+^ T cells were divided into 4 groups as follows: siNeg siRNA group [cells were infected with small interfering RNA (siRNA)-Mate (Shanghai Gene Pharma Co., Ltd., Shanghai, China) for 24 h], 1.2 mM FFA group (cells were infected with siRNA-Mate for 24 h and then treated with 1.2 mM FFA for another 12 h), SDS transient transfection (siSDS) group (cells were transient-transfected with siSDS for 24 h), and siSDS + 1.2 mM FFA group (transfection with siSDS for 24 h and then treated with 1.2 mM FFA for another 12 h). (**A**) Transfection effect. (**B**,**C**) Relative mRNA abundance of *IL-17A* and *FOXP3*. (**D**) ELISA assay for IL-17 concentrations in silenced SDS cells. (**E**,**F**) Analysis of IL-17A and FOXP3 in siSDS cells by flow cytometry. All experiments were repeated 3 times; *n* = 3. A one-way ANOVA with a Duncan correction was used to calculate group comparisons. The data presented are the mean ± SEM; ** *p* ≤ 0.01, * *p* ≤ 0.05.

As illustrated in Figure 3A, the *SDS* expression was successfully silenced. The transcript level of *IL-17A* (*p =* 0.0276) was significantly lower, while the transcript levels of *FOXP3* (*p =* 0.4408) were upregulated in the siSDS + FFA group compared with the FFA group (Figure 3B,C). The ELISA results revealed that the content of IL-17 (*p =* 0.0001) in the siSDS + FFA group was significantly lower compared with the FFA group (Figure 3D). The flow cytometry findings revealed that IL-17A (*p =* 0.0248) expression was lower and FOXP3 (*p =* 0.0001) expression was greater in the siSDS + FFA group (Figure 3E,F).

### 3.4. Effect of ORAI1 on CD4^+^ Th17/Treg

**Figure 4:**
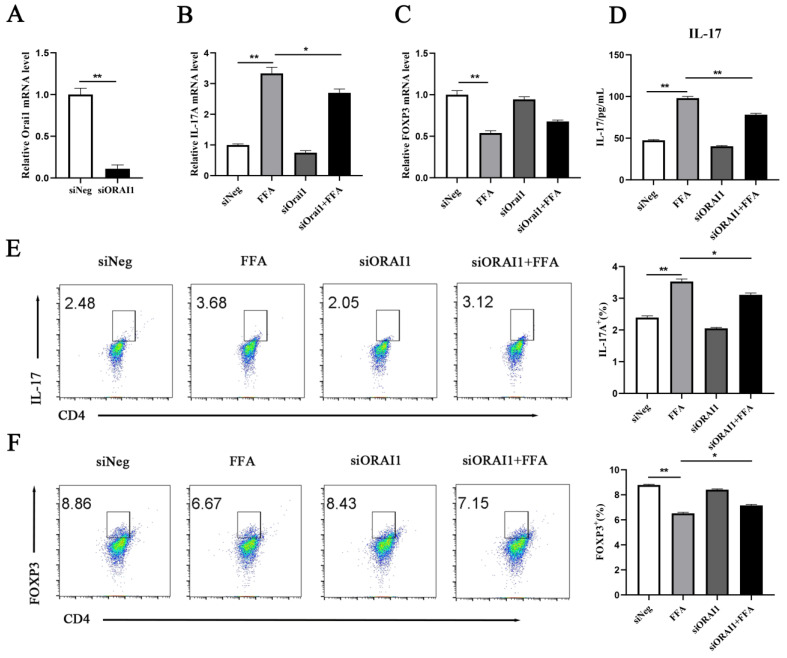
CD4^+^ T cells were divided into 4 groups as follows: siNeg siRNA group [cells were infected with small interfering RNA (siRNA)-Mate (Shanghai Gene Pharma Co., Ltd., Shanghai, China) for 24 h], 1.2 mM FFA group (cells were infected with siRNA-Mate for 24 h and then treated with 1.2 mM FFA for another 12 h), ORAI1 transient transfection (siORAI1) group (cells were transient-transfected with siORAI1 for 24 h), and siORAI1 + 1.2 mM FFA group (transfection with siORAI1 for 24 h and then treated with 1.2 mM FFA for another 12 h). (**A**) Transfection effect. (**B**,**C**) Relative mRNA abundance of *IL-17A* and *FOXP3*. (**D**) ELISA assay for IL-17 concentrations in silenced SDS cells. (**E**,**F**) Analysis of IL-17A and FOXP3 in siORAI1 cells by flow cytometry. All experiments were repeated 3 times; *n* = 3. A one-way ANOVA with a Duncan correction was used to calculate group comparisons. The data presented are the mean ± SEM; ** *p* ≤ 0.01, * *p* ≤ 0.05.

As illustrated in Figure 4A, the *ORAI1* was successfully silenced. The transcript level of *IL-17A* (*p =* 0.0194) in the siORAI1 + FFA group was significantly lower. The transcript level of *FOXP3* (*p =* 0.0416) in the siORAI1 + FFA group was upregulated (Figure 4B,C). The ELISA results revealed that the content of IL-17 (*p =* 0.0001) in the siORAI1 + FFA group was significantly lower compared with the FFA group (Figure 4D). Results from the flow cytometry revealed that IL-17A (*p =* 0.0019) expression was lower and FOXP3 (*p =* 0.0004) expression was greater in the siORAI1 + FFA group (Figure 4E,F).

### 3.5. ORAI1 Regulates CD4^+^ Th17/Treg via Serine Dehydratase (SDS)

**Figure 5:**
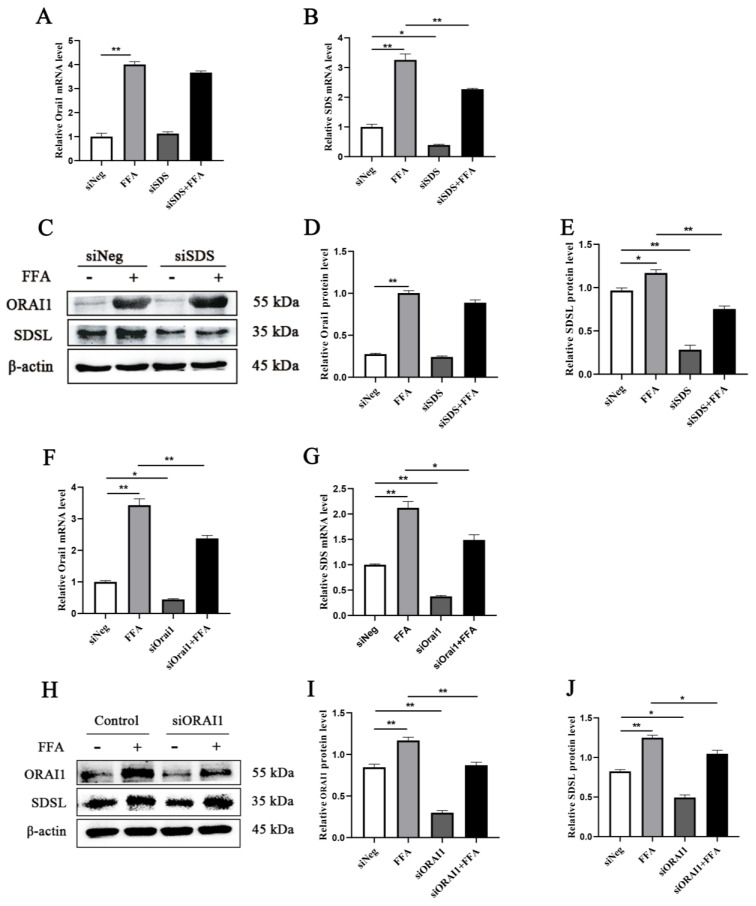
(**A**,**B**) Relative mRNA abundance of ORAI1 and SDS. (**C**) Western blots of ORAI1 and SDSL. (**D**,**E**) Relative abundance protein content of ORAI1 and SDSL. (**F**,**G**) Relative mRNA abundance of ORAI1 and SDS. (**H**) Western blots of ORAI1 and SDSL. (**I**,**J**) Relative abundance protein content of ORAI1 and SDSL. All experiments were repeated 3 times; *n* = 3. A one-way ANOVA with a Duncan correction was used to calculate group comparisons. The data presented are the mean ± SEM; ** *p* ≤ 0.01, * *p* ≤ 0.05.

The silencing ORAI1 by transient transfection results revealed that the transcript level (*p =* 0.0001) and protein abundance (*p =* 0.0005) of ORAI1 were greater in the FFA group; the transcript level (*p =* 0.0001) and protein abundance (*p =* 0.0001) of SDS were greater in the FFA group; the siORAI1 group SDS transcript level (*p =* 0.0019) and protein abundance (*p =* 0.0003) were lower; and the siORAI1 group ORAI1 transcript level (*p =* 0.0247) and protein abundance (*p =* 0.0001) were lower. Compared with the FFA group, the siORAI1 + FFA group transcript level (*p =* 0.0005) and protein abundance (*p =* 0.0008) of ORAI1 were lower, and the transcript level (*p =* 0.0017) and protein abundance (*p =* 0.0069) of SDS were lower (Figure 5A–E). The silencing of SDS by transient transfection results revealed that the transcript level (*p =* 0.0001) and protein abundance (*p =* 0.0001) of ORAI1 were greater in the FFA group; the transcript level (*p =* 0.0001) and protein abundance (*p =* 0.0174) of SDS were greater in the FFA group; and the siSDS group SDS transcript level (*p =* 0.0153) and protein abundance (*p* = 0.0001) were lower. Compared with the FFA group, the expression of ORAI1 in the siSDS + FFA group showed no significant change and the transcript level (*p =* 0.0008) and protein abundance (*p =* 0.0002) of SDS were lower (Figure 5F–J).

## 4. Discussion

A proper adaptation of the mechanisms controlling the immune system after calving when cows have high levels of FFAs ensures that animals have a smooth transition into lactation [24]. Linkages among negative energy balance (NEB), body fat mobilization, and high levels of FFAs with dysfunctional immune responses have been reported in numerous studies [15,25,26]. Further, our data suggest that high FFAs cause CD4^+^ Th17 cell production and reduce CD4^+^ Treg cell production through the ORAI1-sensitive SDS pathway.

CD4^+^ T cells are primarily involved in cellular immunity, controlling and clearing infections, and are an important barrier against infectious diseases [27,28]. These cells are the command center of the body’s immune system, responsible for eliminating and controlling various infections and fighting invading pathogens [29]. Serine is dehydrated and deaminated by the adaptive enzyme serine dehydratase to generate pyruvate [30], which can then be catabolized to acetyl coenzyme A which in turn increases Th17 cell numbers. This sequence of events leads to changes in the production of CD4^+^ Th17/Treg cells.

The endoplasmic reticulum protein STIM1, a single-channel ion-conducting transmembrane protein, interacts with ORAI1 to form the SOCE channel on the cell membrane [31]. ORAI1, a member of the ORAI family of ion channel proteins, is necessary for binding to STIM1 after depletion of intracellular Ca^2+^ stores [32]. Some studies have confirmed that Th17 cell function is greatly influenced by ORAI1, but the specific mechanism in which ORAI1 participates is unknown.

Serine is dehydrated and deaminated under the action of serine dehydratase to generate pyruvate. Pyruvate can generate acetyl-CoA under the action of citrate lyase, and acetyl-CoA stimulates Th17 cell function, thus making CD4^+^ Th17/Treg imbalanced [11,30]. The fact that ORAI1 silencing led to downregulation of serine dehydratase gene and protein expression was noteworthy because the silencing of serine dehydratase did not alter ORAI1 expression. Thus, these data suggest that ORAI acts upstream of SDS to regulate the balance of CD4^+^ Th17/Treg [15].

## 5. Conclusions

Overall, our findings point to the ORAI1-sensitive serine dehydratase pathway as a responder to high FFA stimulation, which then contributes to the CD4^+^ Th17/Treg imbalance. Thus, together, the data indicate that serine dehydratase may serve as a therapeutic target to alleviate high-FFA-induced inflammatory diseases such as ketosis. Although in vitro data revealed that ORAI1 affects the CD4^+^ Th17/Treg imbalance in vivo by regulating serine dehydratase, the applicability of these data to transition cows is still unknown.

## Figures and Tables

**Table 1:** Basic features of healthy and ketosis cows used in the present study.

	Control (*n* = 6)		High-FFA (*n* = 6)	
Item	Median	Interquartile Range	Median	Interquartile Range
BW (kg)	645	640, 649	670	663, 693
Milk yield (kg/d)	37.05	34.1, 38.0	39.6	38.2, 40.4
DMI	20.85	19, 23.4	15.35	14.7, 15.7
Serum glucose (mM)	3.51	2.93, 3.85	2.23	1.99, 2.57
Serum NEFA (mM)	0.38	0.26, 0.53	1.34	1.07, 1.58
Milk fat (%)	4.03	3.14, 4.8	4.67	4.02, 5.64
Protein (%)	3.57	3.05, 4.06	3.21	2.86, 3.72

**Table 2:** Sequences of primers used for real-time PCR amplification.

Gene	GenBank Number	Primer (5′ to 3′)	Length (bp)
*GAPDH*	NM_001034034.2	Forward: GTCTTCACTACCATGGAGAAGG	197
		Reverse: TCATGGATGACCTTGGCCAG	
*ACTB*	NM_173979.3	Forward: GCTAACAGTCCGCCTAGAAGCA	403
		Reverse: GTCATCACCATCGGCAATGAG	
*SDS*	NM_001075662.1	Forward: GCCTCTTGTGCGGAGTGGTTC	109
		Reverse: GCCTTGGTGGAAGCGTGGAAG	
*IL-17A*	NM_001008412.2	Forward: CACAGCATGTGAGGGTCAAC	101
		Reverse: GTGGAGAGTCCAAGGTGAGG	
*FOXP3*	NM_001045933.1	Forward: TGGTGCAATCTCTGGAGCAA	116
		Reverse: GTCAGATGATGCCGCAGATG	
*ORAI1*	NM_001099002.1	Forward: TTTGCCGTCCACTTCTAC	285
		Reverse: CCTCTTTCCTCCACTTTCT	

## Data Availability

The original contributions presented in this study are included in the article/Supplementary Materials. Further inquiries can be directed to the corresponding author.

## Supplementary Materials

The following supporting information can be downloaded at: https://www.mdpi.com/article/10.3390/ani15030388/s1, Figure S1: Isolation of CD4^+^ T cells from peripheral blood of healthy and high FFA cows; Figure S2: Isolation of CD4^+^ T cells from peripheral blood of healthy and high FFA cows.

## Abbreviations

FFAs	free fatty acids
NEB	negative energy balance
ORAI1	ORAI calcium release-activated calcium modulator 1
SOCE	store-operated calcium entry
STIM1	stromal interaction molecule 1
SDS	serine dehydratase
GAPDH	glyceraldehyde-3-phosphate dehydrogenase
