# Supplementary material for: Calcium Release-Activated Calcium Modulator ORAI1-Sensitive Serine Dehydratase Regulates Fatty Acid-Induced CD4+ Th17/Treg Imbalance in Dairy Cows
Source: Animals (Basel). 2025 Jan 30;15(3):388. doi: 10.3390/ani15030388 (PMC11815743; doi:10.3390/ani15030388)
Supplement: Supplementary file 1 [file animals-15-00388-s001.zip › animals-3355580-supplementary.pdf]

Supplementary Figure S1

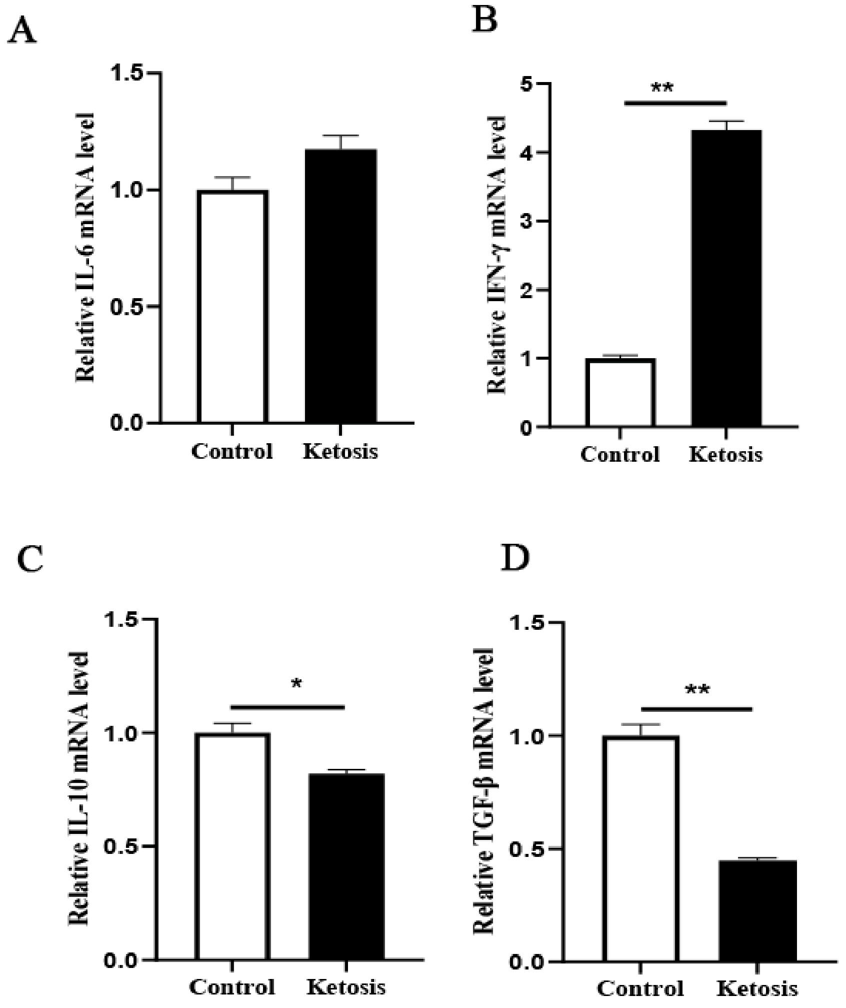

Supplementary Figure S1. Isolation of CD4+ T cells from peripheral blood of healthy and high FFA cows. (A-D) Relative levels of mRNA for IL-6、IFN-γ、IL-10 and TGF-β. Independent samples t-test for comparison. The data presented are the mean ± SEM; $^{**}P \leq 0.01$, $^{*}P \leq 0.05$.

Supplementary Figure S2

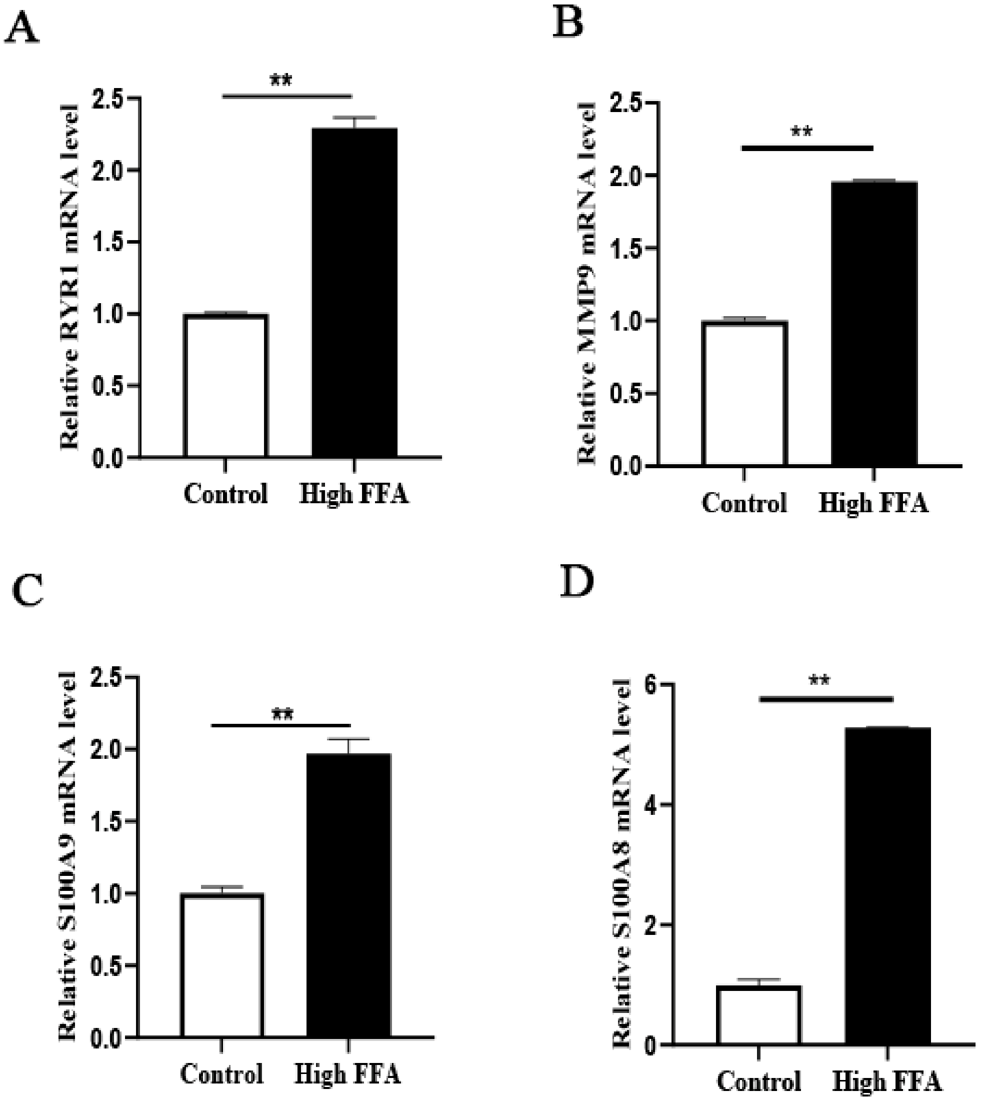

Supplementary Figure S2 Isolation of $CD4^+$ T cells from peripheral blood of healthy and high FFA cows. (A-D) Relative levels of mRNA for RYR1、MMP9、S100A9 and S100A8. Independent samples t-test for comparison. The data presented are the mean ± SEM; **P ≤ 0.01 .
